# Gastrointestinal motility following thoracic surgery: the effect of thoracic epidural analgesia. A randomised controlled trial

**DOI:** 10.1186/s12871-017-0427-y

**Published:** 2017-10-16

**Authors:** Argyro Zoumprouli, Aikaterini Chatzimichali, Stamatios Papadimitriou, Alexandra Papaioannou, Evaghelos Xynos, Helen Askitopoulou

**Affiliations:** 1 Anaesthetic Department, St. George’s University Hospitals NHS Foundation Trust, Blackshaw Road, London, SW17 0QT UK; 2 Anaesthetic Department, University Hospital of Heraklion, Stavrakia, 71110 Heraklion, Greece; 3 Asklipeion Kritis, 8 Zografou str, Heraklion, Greece; 4 Colorectal Surgery, Creta interclinic, Heraklion, Crete Greece; 5 Faculty of Medicine, University of Crete, Stavrakia, 71003 Heraklion, Greece

**Keywords:** Perioperative medicine and outcome, Thoracic epidural analgesia, Postoperative Ileus, Oro-ceacal transit time, Ropivacaine, Morphine

## Abstract

**Backgrounds:**

Impairment of gastrointestinal (GI) motility is an undesirable but inevitable consequence of surgery. This prospective randomised controlled study tested the hypothesis that postoperative thoracic epidural analgesia (TEA) with ropivacaine or a combination of ropivacaine and morphine accelerates postoperative GI function and shortens the duration of postoperative ileus following major thoracic surgery compared to intravenous (IV) morphine.

**Methods:**

Thirty patients scheduled for major thoracic surgery were randomised to three groups. All patients had bowel motility assessments 1 week preoperatively. All patients received general anaesthesia. Group Ep-R received TEA with ropivacaine; group Ep-RM received TEA with ropivacaine and morphine and group IV-M received IV morphine via patient controlled analgesia pump (PCA). Bowel motility was assessed by clinical examination in addition to oro-ceacal transit time (OCTT) on the first and third postoperative days and colonic transit time (CTT).

**Results:**

Overall the OCTT demonstrated a 2.5-fold decrease in bowel motility on the first postoperative day. The OCTT test revealed statistically significant differences between all groups (Ep-R vs Ep-RM, *p* = 0.43/Ep-R vs IV-M, *p* = 0.039 / Ep-RM vs IV-M, *p* < 0.001). Also, very significant differences were found in the OCCT test between days (Ep-R vs Ep-RM, p < 0.001/Ep-R vs IV-M, p < 0.001 / Ep-RM vs IV-M, *p* = 0.014). There were no significant differences in the CTT test or the clinical signs between groups. However, 70% of the patients in the Ep-R group and 80% in the Ep-RM group defecated by the third day compared to only 10% in the IV-M group, (*p* = 0.004).

**Conclusions:**

Objective tests demonstrated the delayed motility of the whole GI system postoperatively following thoracic surgery. They also demonstrated that continuous epidural analgesia with or without morphine improved GI motility in comparison to intravenous morphine. These differences were more pronounced on the third postoperative day.

**Trial registration:**

ISRCTN number: 11953159, retrospectively registered on 20/03/2017.

## Background

Impairment of gastrointestinal (GI) motility is an undesirable but inevitable consequence of abdominal or other surgery that delays recovery and may prolong hospital stay [1, 2]. This effect, referred to as postoperative ileus (POI), is defined as a transient disruption of the normal coordinated movements of the gut preventing the effective transit of the intestinal contents to varying degrees [3]. The aetiology of this functional, non-mechanical obstruction of the bowel is complex, primarily associated with the surgical stress response, and also with activated reflex arcs of sympathetic activity to surgical injury and postoperative pain [4]. The stress response initiates a cascade of acute physiological, metabolic and inflammatory events that start with the initiation of general anaesthesia and last 3 to 4 days postoperatively, depending on the type of the anaesthetic and postoperative analgesia techniques [5, 6].

TEA can enhance bowel motility by producing a sympathectomy that leaves the parasympathetic innervation of the gut unopposed, and also by providing pain relief, thus diminishing the systemic stress response [7–12].

Postoperative analgesia with IV morphine has a negative effect on bowel propulsion, through activation of the peripheral μ-receptors of the gut [13]. Further, TEA with opioids versus combination of opioids and local anaesthetics (LAs) has conflicting effects on the activation of the sympathetic response [14]. The positive effect of TEA on gut motility becomes clearer by a multimodal standardised recovery programme [1, 11, 13, 15] and extending TEA for longer than 2 days [7, 9].

Most studies of gastrointestinal dysfunction use clinical indicators to assess POI. However, clinical indicators such as the time to first flatus or stools, correlate poorly with the recovery of the GI function, as they may mirror rectal emptying [16, 17]. More objective measures of GI function are the OCTT measured by lactulose H_2_-breath test, a non-invasive method based on the metabolic release of H_2_ in the human colon and the CTT of radiopaque markers determined by abdominal X-rays at specified times [18, 19].

The present prospective randomised controlled study tested the hypothesis that postoperative thoracic epidural analgesia with ropivacaine or a combination of ropivacaine and morphine accelerates postoperative GI function and shortens the duration of POI following major thoracic surgery compared to IV morphine. The primary outcome measures used were the OCTT, the CTT and the presence of bowel sounds, flatus and stools, while a secondary outcome measure was the visual analogue (VAS) pain score.

## Methods

This prospective randomised controlled study, was approved by the ethics committee of the University Hospital of Heraklion, Greece (No 3197, 19 March 2002). The study was performed between March 2002 and January 2009 and was registered retrospectively (20/03/2017) at ISRCTN registry system with registration number: ISRCTN11953159. All patients provided written informed consent. Only patients undergoing major thoracic surgery (thoracotomy) were recruited in order to avoid the confounding direct effect of intra-abdominal surgery on the GI system. Furthermore, a standardised postoperative recovery regimen of feeding, ambulation and pain score targets was followed, with the purpose of controlling and optimising factors that affect the recovery of GI function. The exclusion criteria were diabetes mellitus, history of chronic pain, drug/alcohol dependence, corticosteroid use, treatment with drugs known to affect GI motility, inflammatory bowel disease, previous bowel surgery, previous history of abdominal radiation, morphine or local anaesthetic allergy, ASA physical status > III, age younger than 30 or older than 85 years, presence of contraindications to insertion of an epidural catheter and severe renal and liver disease. All patients had a history of normal bowel habits. Eligible patients were randomly assigned to three groups by opening opaque sealed envelops in the anaesthetic room prior to surgery. The groups were pre-determined by a computer-generated list of random numbers (block of 6 with 1:1:1 allocation).

### Preoperative period

All patients underwent standard preoperative assessment and received teaching on how to score pain and to report side effects. In addition, they were informed about postoperative tests, feeding and early ambulation, as well as postoperative visits from different teams. 1 h before surgery, all patients received premedication with intramuscular 0.07 mg.kg^−1^ midazolam. On arrival to the anaesthetic room, each patient was randomised in one of three analgesic groups: group Ep-R, ropivacaine epidurally, group Ep-RM a combination of ropivacaine and morphine epidurally and group IV-M IV morphine by PCA. In groups Ep-R and Ep-RM a thoracic epidural was performed before induction of general anaesthesia between the levels T_5–9_ using loss of resistance technique with an 18G Tuohy needle and a 20G epidural catheter was inserted 3–5 cm into the epidural space. After negative aspiration of blood and cerebrospinal fluid, a test dose of 3 ml of lidocaine 2% containing adrenaline 5 mcg.ml^−1^ was injected through the catheter.

### Intra-operative period

General anaesthesia was induced in all patients with intravenous fentanyl 1.5–2 mcg.kg^−1^, propofol 1.5–3 mg.kg^−1^ followed by rocuronium 0.6 mg.kg^−1^ or cis-atracurium 0.1 mg.kg^−1^. Intubation of the trachea was performed using a Robertshaw left-sided double lumen endotracheal tube. Following the placement of the patient in the lateral right or left decubitus position, the correct tracheal and bronchial tube position was confirmed by a fibreoptic bronchoscope.

Anaesthesia was maintained with an oxygen/air mixture and either propofol continuous infusion or sevoflurane administration. 20 min before the surgical incision group Ep-R received the first epidural bolus of 5 ml of 0.5% ropivacaine (5 mg.ml^−1^), group Ep-RM received 5 ml of 0.5% ropivacaine and 3 mg of morphine in 8 ml of sterile normal saline 0.9%, and group IV-M received an IV bolus of 0.05 mg.kg^−1^ morphine. Neuromuscular blockade was maintained with rocuronium or cisatracurium boluses, as needed and monitored with a train of four stimuli from a peripheral nerve stimulator. Intraoperative monitoring also included pulse oximetry, electrocardiography, end-tidal CO_2_, invasive arterial pressure and urinary output. All patients were placed on a water-warming mattress. Blood pressure and heart rate were maintained at ±20% of preoperative baseline values throughout the operation with the use of phenylephrine boluses of 40 mcg, as required. Patients received further epidural or IV boluses according to analgesic requirements and group allocation. Intraoperative blood loss (from suction and weighted surgical dressings) was recorded and replaced by crystalloids, colloids and blood products according to individual needs and departmental policy.

At the end of surgery neuromuscular blockade was reversed using neostigmine 2.5 mg in combination with glycopyrrolate 0.5 mg, and the double lumen tube was removed. Patients were then transferred to the post-anaesthesia care unit, where they remained for at least 2 h for monitoring and clinical observation. Invasive arterial pressure monitoring was removed before patients’ transfer to the surgical ward. The same surgical team performed the surgery on all patients and did not take part in the collection of study data.

### Postoperative analgesia regimes

At the end of the surgical procedure, an epidural or an IV infusion was started according to group allocation. Group Ep-R received a continuous epidural infusion of ropivacaine 0.2% (2 mg.ml^−1^) at a rate of 5–8 ml.h^−1^, with boluses of 2 ml of the same solution and a 20-min lockout interval via a PCA pump. Group Ep-RM received a continuous epidural infusion of ropivacaine 0.15% (1.5 mg.ml^−1^) with morphine 0.05 mg.ml^−1^ at a rate of 5–7 ml.h^−1^, with boluses of 2 ml of the same solution and a 20-min lockout interval via a PCA pump. Group IV-M received a continuous IV infusion of morphine of 1 mg.ml^−1^.h^−1^, with boluses of 0.5–1 mg and a 15-min lockout interval also via a PCA pump.

Both patients and the research team were double blinded for the epidural groups and unblinded for the group IV-M. Only the Acute Pain Team was aware of the solutions administered and was allowed to give extra boluses accordingly, if and when needed, so that analgesia was titrated to a VAS score at rest of <5 (on a 10-point scale, where 0 = no pain, 10 = worst possible pain). The level of epidural block was evaluated by loss of pain sensation to pinprick. No other analgesics were used. The analgesic regimes were continued until at least the third postoperative day.

### Postoperative evaluation of GI regime

On the first postoperative morning, all patients were started on a standardised low-fat diet. Mobilisation was commenced from the first postoperative day. The surgical, acute pain and research teams visited the patients independently twice a day to optimise and monitor the postoperative course and care. The surgical team decided patients’ discharge.

In all patients GI motility was assessed on the first and third postoperative days by two objective tests: (a) OCTT using the hydrogen (H_2_) breath test and (b) CTT using radiopaque markers, and also by subjective tests: (a) the passage of flatus (b) defecation and (c) the presence of bowel sounds.

The OCTT was evaluated by the lactulose - H_2_ breath test, a non-invasive method widely used for quantifying the OCTT. The OCTT test measures the time (in minutes) taken for lactulose to reach the caecum. Lactulose is a synthetic disaccharide that cannot be absorbed in humans and therefore passes unchanged to the colon where it is fermented by bacteria. The H_2_ produced by fermentation, passes into the blood stream and therefore can be measured in the exhaled breath from the lungs. The OCCT the point at which H_2_ is increased in the exhaled breath.

Patients were advised to fast for at least 12 h prior to the breath test and to avoid fibre rich food for 24 h. The day of the test, the subjects were fasting and the exhaled H_2_ concentrations in breath were measured in parts per million (ppm) by an electrochemical detector (Lactoscreen, Hoek Loos, Schiedam, The Netherlands) following an oral load of 10 g lactulose (diluted in 200 ml of water) at time 0 and every 15 min thereafter for up to 4 h, or when the subject reached an increment of 10 ppm H_2_. The detector was calibrated using samples of room air (undetectable H_2_) and a standard gas mixture containing 100 ppm H_2_ (automated process). Before the test the patient was rested for at least 1 min. Breath samples were collected by aspirating aliquots of end-expiratory air into 20 ml plastic syringes with a three-way stopcock. The measurements were conducted in the sitting position in a quiet, well-ventilated room, without physical activity during the test. The OCTT test was performed at three times: (a) 1 week before surgery (OCTT_0_), (b) on the first postoperative morning (OCTT_1_) and (c) on the third postoperative morning (OCTT_3_).

The CTT was evaluated by plain abdominal radiographs taken 4 days after the ingestion of 20 radiopaque markers consisting of 1–2 mm of hollow radiopaque polyethylene tubing. The markers were ingested 1 week before surgery (CCT_0_) and on the first postoperative morning (CCT_1_). The measurement of the total number of markers retained after 96 h assessed the progression of radiopaque markers along the large bowel, giving an indication of CTT.

#### Data collection

OCTT was recorded 1 week and the CTT 4 days preoperatively. Patient demographics were also recorded during the preoperative period. The total intraoperative amounts (in mg) of epidural ropivacaine and epidural or IV morphine were recorded, as well as the level of the thoracic epidural catheter placement, the duration of surgery, blood loss, and intraoperative fluid replacement.

During the postoperative period, both the acute pain and the research teams collected data independently until patient discharge. Postoperative colonic motility was evaluated by the OCTT and CTT tests as well as by the clinical signs of the first passage of flatus, faeces and the first presence of bowel sounds. The amount (in mg) of epidural ropivacaine and epidural or IV morphine administered, the VAS pain scores and the upper and lower sensory levels (pinprick sensation) of the epidural blockade were recorded, as well as blood pressure, heart rate and respiratory rate measurements. The VAS scores were measured at rest (VAS rest) and on mobilisation (VAS dynamic), on the first, second, and third postoperative days. The patients were monitored for side effects such as nausea, vomiting, pruritus, sedation, motor or sensory block, hypotension, bradycardia and respiratory depression and treated appropriately.

#### Statistical analysis

A small pilot study demonstrated that OCTT time was increased by 270% in patients receiving continuous IV infusion of morphine 1 ml.h^−1^ and PCA boluses. Based on the assumption that a 30% difference in the time of recovery of the GI function (assessed by OCTT) between the epidural groups and the IV morphine group was of clinical significance, a power analysis estimated that nine patients per group were needed to provide 80% power and 0.05 alpha error.

Data are presented as the mean ± SD, numbers (%), or median (interquartile range). Statistical analyses were performed using the SPSS software version 24 (IBM Corp., USA). Between-group differences for the OCTT test were analysed using General Linear Model (GLM) – repeated measures. One-way ANOVA (3 groups comparison) for parametric variables or the Kruskal–Wallis test for non-parametric variables were also used. The Fisher exact test was used to analyse categorical variables, where appropriate. Pearson’s and Spearman’s rho rank correlation coefficients were used to assess the degree of association between variables. Values for *p* < 0.05 were considered statistically significant.

## Results

**Fig. 1:**
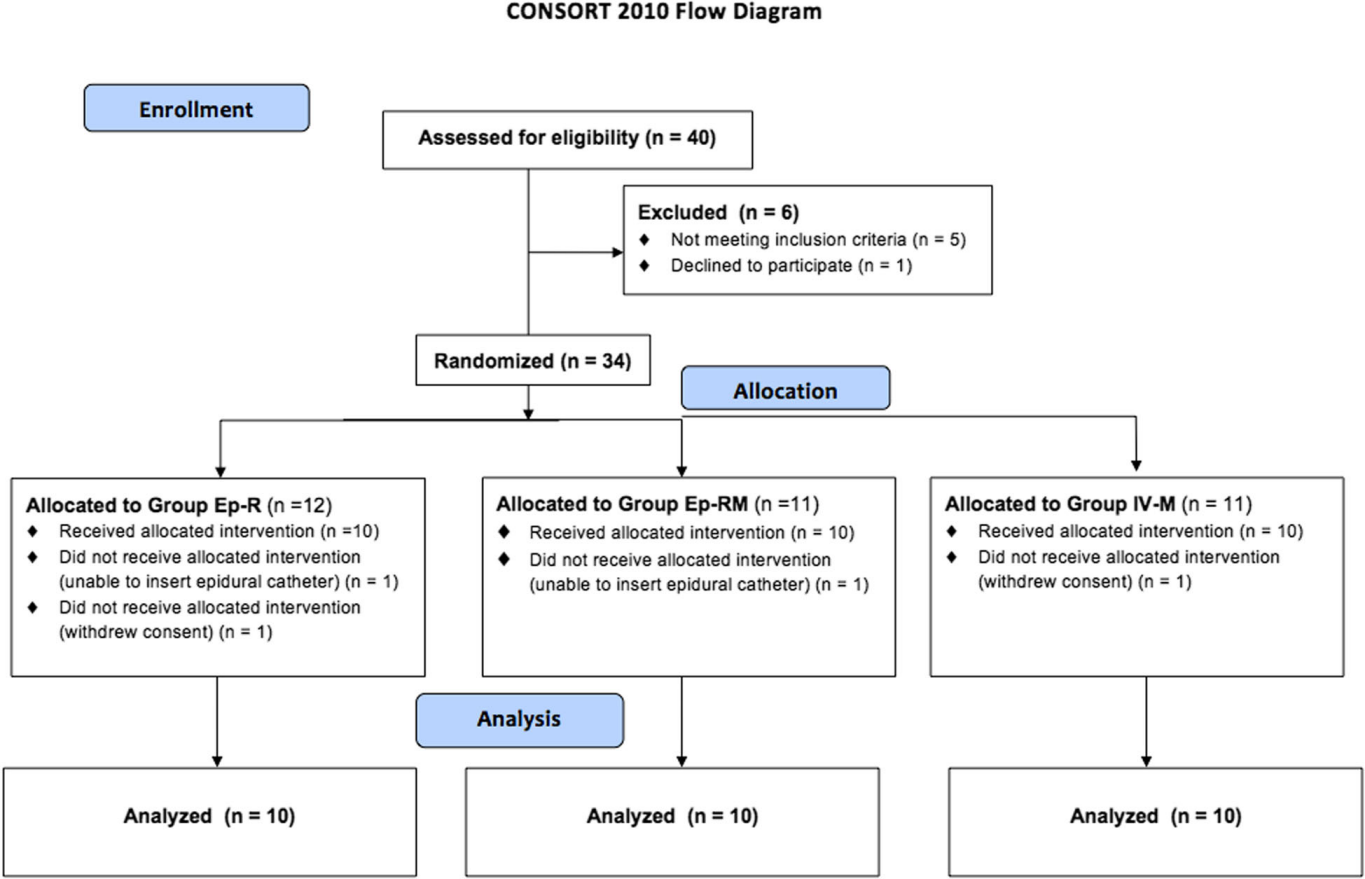
Consolidating Standards of Reporting Trials (CONSORT) diagram

Of the 40 patients enrolled, 34 were randomised (Fig. 1), and 30 completed the study (3 women and 27 men), 31 to 82 years old, ASA physical status III.

The three groups did not differ significantly regarding patient demographics, type and duration of surgery, intraoperative blood loss and fluid replacement (Table 1). Intraoperatively, none of the patients had obvious vagal damage. The epidural catheter was inserted between T_5_-T_9_ levels. The upper sensory level of the block in the epidural groups had a median at T_4_ (T_3_-T_5_), while the lower sensory level had a median at T_9_ (T_8_-T_11_) (Table 1). No patient experienced postoperative motor blockade.

**Table 1:** Demographic and perioperative data

	Group Ep-R	Group Ep-RM	Group IV-M	*p*-value
Age (yr)	63 ± 10	59 ± 13	61 ± 11	0.79
Weight (kg)	76 ± 14	75 ± 12	79 ± 10	0.71
Height (cm)	176 (175–182)	179.5 (178–184)	178.50 (176–182)	0.65
Estimated blood loss (ml)	200 (200–600)	475 (100–600)	200 (100–500)	0.83
Duration of surgery (min)	153 ± 53	175 ± 84	188 ± 82	0.58
Fluids Crystalloids (ml.kg^−1^)	26 ± 16	37 ± 23	26 ± 13	0.31
Fluids Colloids (ml.kg^−1^)	10 ± 7	14 ± 11	10 ± 11	0.55
Epidural details				
Level of epidural catheter	T_6_ (*n* = 2)	T_5_ (n = 1)	NA	–
	T_7_ (*n* = 4)	T_6_ (*n* = 4)		
	T_8_ (*n* = 4)	T_7_ (*n* = 4)		
		T_9_ (*n* = 1)		
Upper Sensory level	T_2_ (*n* = 1)	T_3_ (*n* = 2)	NA	–
	T_3_ (*n* = 2)	T_4_ (*n* = 5)		
	T_4_ (*n* = 4)	T_5_ (*n* = 3)		
	T_5_ (*n* = 1)			
	T_6_ (*n* = 1)			
Lower sensory level	T_8_ (*n* = 4)	T_8_ (*n* = 3)	NA	–
	T_9_ (*n* = 1)	T_9_ (*n* = 3)		
	T_11_ (*n* = 2)	T_10_ (*n* = 3)		
	T_12_ (*n* = 1)	T_11_ (*n* = 1)		
	L_3_ (*n* = 1)			
Total sensory levels blocked	7 (5–8)	6 (5–6)	NA	0.13
Type of surgery				
Lobectomy	*n* = 8	*n* = 6	*n* = 8	–
Mass resection	*n* = 2	*n* = 4	*n* = 1	–
Thoracic wall tumour	–	–	*n* = 1	–
Side effects				–
Orthostatic hypotension	*n* = 2	*n* = 1	–	
Nausea	–	*n* = 1	*n* = 1	
Pruritus	–	*n* = 1	*n* = 2	
Drowsiness	–		*n* = 1	
SBP_1_	119 ± 16	128 ± 18	129 ± 15	0.361
DBP_1_	69 ± 10.31	73 ± 10	72 ± 12	0.393
HR_1_	83 ± 7	78 ± 7	83 ± 11	0.431
SBP_3_	122 ± 11	124 ± 24	128 ± 16	0.362
DBP_3_	69 ± 7	69 ± 8	77 ± 22	0.679
HR_3_	84 ± 5	83 ± 14	78 ± 32	0.827

Data are expressed as mean ± SD, median (interquartile range) or absolute values. *p* values <0.05 represent statistically significant results. (*Ep-R* Epidural Ropivacaine, *Ep-RM* Epidural Ropivacaine and Morphine, *IV-M* Intravenous Morphine, *SBP*_*1,3*_ systolic blood pressure on 1st and 3rd postoperative day respectively, *DBP*_*1,3*_ diastolic blood pressure on 1st and 3rd postoperative day respectively, *HR*_*1,3*_ heart rate on 1st and 3rd postoperative day respectively

**Fig. 2:**
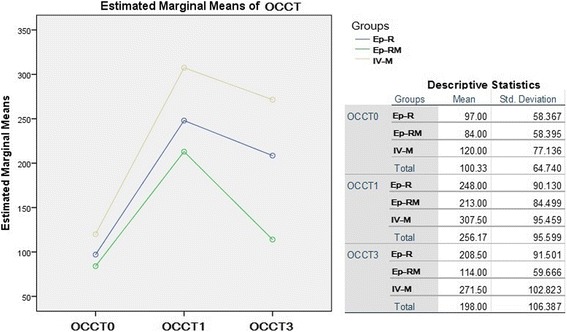
GLM Repeated Measures Results Data are expressed as mean ± SD. (Ep-R = Epidural Ropivacaine, Ep-RM = Epidural Ropivacaine and Morphine, IV-M = Intravenous Morphine, OCTT0 = oro-ceacal transit time preoperatively, OCTT1 = oro-ceacal transit time first postoperative day, OCTT3 = oro-ceacal transit time third postoperative day

The preoperative OCTT and CCT tests were not different between the three groups (*p* = 0.44 and *p* = 0.28 respectively) (Table 2). The total intraoperative doses of ropivacaine between the two epidural groups did not differ statistically (*p* = 0.739), in contrast to morphine that was administered via two different routes (Table 3). On the whole, the OCTT demonstrated a 2.5-fold decrease of bowel motility on the first postoperative day (OCTT_0_ 100 ± 64.73 vs OCTT_1_ 256.16 ± 95.59). The GLM analysis of the OCTT measures revealed a significant effect between groups (F = 408.192, *p* < 0.001) and also between days (F = 30.126, p < 0.001). More precisely, there was a statistically significant difference in OCCT measurements between all groups (Ep-R vs Ep-RM, *p* = 0.43/Ep-R vs IV-M, *p* = 0.039/EpRM vs IV-M, *p* < 0.001), as well as between days (Ep-R vs Ep-RM *p* < 0.001 / Ep-R vs IV-M, *p* < 0.001/Ep-RM vs IV-M, *p* = 0.014). No interaction was found between groups and OCTT tests (Fig. 2).

**Table 2:** GI motility evaluation results

	Group Ep-R	Group Ep-RM	Group IV-M	*p*-value
Radiopaques_0_ (n)	2 (1–3)	1 (0–3)	5 (1–18)	0.28
Radiopaques_4_ (n)	11 (2–18)	12.5 (1–18)	17 (10–19)	0.41
Defecation_1_ (%)	10%	0%	0%	0.35
Defecation_3_ (%)	70%	80%	10%	**0.004**

Values are expressed as median (interquartile range), or proportions as indicated. *p* values <0.05 represent statistically significant results (bold). (*Ep-R* Epidural Ropivacaine, *Ep-RM* Epidural Ropivacaine and Morphine, *IV-M* Intravenous Morphine, *Radiopaques*_*0*_ number of radiopaques peoperatively, *Radiopaques*_*4*_ number of radiopaques postoperatively)

**Table 3:** Total amount of ropivacaine and morphine administered in the three groups

	Group Ep-R	Group Ep-RM	Group IV-M	*p*-values
Ropivacaine (mg)				(Ep-R vs Ep-RM)
Intra-operatively	106 (90–125)	115 (100–135)	–	0.739
1st post-op day	390 ± 112	338 ± 112	–	0.353
3st post-op day	1014 (680–1328)	823 (673–861)	–	0.123
Morphine (mg)				(Ep-R vs IV-M)
Intra-operatively	–	3.5 (3–4)	8 (5–10)	**0.029**
1st post-op day	–	11 ± 1.5	52 ± 18	**<0.001**
3st post-op day	–	28 (26–32)	114 (108–125)	**<0.001**

Data are expressed as mean ± SD or median (interquartile range). *P* values <0.05 represent statistically significant results (bold). (*Ep-R* Epidural Ropivacaine, *Ep-RM* Epidural Ropivacaine and Morphine, *IV-M* Intravenous Morphine)

There was no significant difference between groups in the migration of radiopaque markers (Table 2), as well as in the presence of bowel sounds and the passage of flatus on the first or third postoperative days. However, 70% of the patients in the Ep-R group and 80% in the Ep-RM group defecated by the third day compared to only 10% in the IV-M group, a statistically significant finding (*p* = 0.004) (Table 2). Spearman’s rank correlation coefficient was used to explore the relationship between defecation and presence of radiopaques on the first postoperative day (Rho = −0.13, *p* = 0.48 and third postoperative day (Rho = −0.38, *p* = 0.04).

The total amount of ropivacaine administered by the first and third postoperative days was not different between the epidural groups, in contrast to the amount of morphine that was much higher in the IV-M group (Table 3). The correlation between total ropivacaine dose and OCTT was explored and was not significant (Table 4). Throughout the postoperative period, all patients had adequate analgesia, with no significant differences between groups for the VAS pain scores at rest or during ambulation (Table 5). The non-parametric Spearman coefficient showed no correlation between VAS rest or dynamic and the OCTT values of the first and third postoperative days.

**Table 4:** Spearman’s rho rank correlation coefficient (Rho) between total mg of ropivacaine administered epidurally and OCTT

Ropivacaine (mg)	OCTT_1_	OCTT_3_
Intraoperatively	Rho = −0.49 *p* = 0.005	Rho = −0.31 *p* = 0.093
1st postoperative day	Rho = −0.32 *p* = 0.083	Rho = −0.4 *p* = 0.03
3rd postoperative day	Rho = −0.29 *p* = 0.12	Rho = −0.43 *p* = 0.02

Values close to +1 or −1 indicate strong association between variables. *p* values <0.05 represent statistically significant results. *OCTT*_*1*_ oro-ceacal transit time the first postoperative day, *OCTT*_*3*_ oro-ceacal transit time the third postoperative day

**Table 5:** VAS pain scores (0–10) at rest and during ambulation on the first (VAS_1_) and third (VAS_3_) postoperative days

	Group Ep-R	Group Ep-RM	Group IV-M	*p*-value
VAS_1_ rest	1 (0–4)	1 (0–2)	2 (1–3)	0.35
VAS_3_ rest	2 (1–4)	0 (0–1)	2 (2–4)	0.09
VAS_1_ dynamic	5 (3–8)	4 (3–5)	5 (3–7)	0.71
VAS_3_ dynamic	6 (5–6)	4 (3–5)	5 (4–6)	0.22

Values are expressed as median (interquartile range). *p* values <0.05 represent statistically significant results. (*Ep-R* Epidural Ropivacaine, *Ep-RM* Epidural Ropivacaine and Morphine, *IV-M* Intravenous Morphine)

Postoperative hemodynamic data between groups were not significantly different (Table 1). All patients were discharged on the sixth postoperative day as per local surgical protocol.

## Discussion

The key finding of the present controlled randomised study was that all patients undergoing major thoracic surgery had significantly reduced GI motility both on the first and the third postoperative days regardless of the postoperative analgesic technique used. GI motility recovered faster in patients who received TEA with ropivacaine and morphine in a standardised recovery programme compared to TEA with ropivacaine alone or IV morphine. The objective OCTT test of GI mobility revealed that although the effect of the TEA was beneficial from the first postoperative day, it became more pronounced on the third postoperative day.

POI is an important common clinical problem following abdominal surgery [20, 21], but also extra-abdominal procedures [2, 22] or noxious stimuli [23]. Since the early nineteenth century, it has been known that stressful, centrally acting stimuli have marked effects on the GI tract. Incision of the peritoneum inhibits the migrating myoelectric complex (MMC) activity, while prolonged inhibition is present after bowel manipulation [23]. To avoid the direct effect on the GI tract from bowel manipulation and local bowel inflammation factors, we only enrolled patients scheduled for major thoracotomy.

All general anaesthetics and short-acting opioids used for induction and maintenance of anaesthesia depress GI motility, but their effects are not prolonged or significant [1]. It is well established that the systemic or epidural administration of opioids decreases gastric emptying, affects MMC activity of the small bowel and decreases propulsive waves in the colon [1, 23, 24]. Equally, the epidural administration of local anaesthetics blocks afferent and efferent inhibitory reflexes, increases splanchnic blood flow and exhibits anti-inflammatory effects via the systemic absorption [10]. However, although extensive epidural blockade with LAs can prevent the endocrine and metabolic responses to surgery in the pelvis and lower limbs, in thoracic surgery it is not possible to block completely the stress response even with a block up to the C_6_ dermatome [5].

Data about the epidural administration of LAs and the combination of LAs and opioids on GI function following non-abdominal surgery are sparse. The only study of mid-TEA with fentanyl and bupivacaine following thoracotomy is that of Guha et al., who demonstrated reduced gastric emptying, using the paracetamol absorption technique [6]. Several studies and reviews have concluded that the epidural administration of local anaesthetics in patients undergoing different types of abdominal surgery provides a faster recovery of the GI hypomotility compared with the systemic or epidural administration of opioids without any increased risk of GI complications [3, 9, 11, 12, 14, 20]. However, the study by Liu et al. showed no difference between epidural LAs and the combination of epidural LAs and opioids following colon surgery, but both groups showed faster return of GI function than those with systemic or epidural opioids [3]. This was one of the first studies to include a standardised recovery programme to control non-analgesic factors that may influence the rate of GI recovery. Furthermore, a systematic review by Shi et al. of the effect of thoracic epidural analgesia vs. systemic analgesia on the recovery of GI function following GI surgery presented evidence that TEA (compared to systemic analgesia) improved the recovery of GI function after GI procedures even when the analgesic regime included opioids in combination with LAs [9]. In addition, it showed that for the TEA to have a beneficial effect on the motility of the gut, it should be administered for at least 2–3 days after surgery [9]. These findings are in agreement with our findings, which showed that both TEA groups with ropivacaine or ropivacaine and morphine were superior to IV morphine in particular on the third postoperative day.

The present study also demonstrated that patients receiving TEA with ropivacaine and morphine had faster GI recovery compared to those receiving TEA with ropivacaine alone. This finding can be explained by the effect of epidural morphine on the central nervous system, where opioids suppress hypothalamic and pituitary hormone secretion. Although the primary sites of opioid inhibition of the GI function are on the μ-receptors in the peripheral nervous system [24], the central analgesic and hormonal effects of opioids may also be important. It is possible that epidural morphine decreases the activity of the adrenocortical system and blocks the stress response directly at the hypothalamic level, while epidural LAs cannot block it completely. The present study did not explore other outcomes that may be related to the overall stress response [5, 25].

Perioperative factors of importance in the control of postoperative recovery as well as a multimodal approach to postoperative care should be considered in all studies of the effects of postoperative analgesia on surgical outcomes [12, 20]. In this study, factors known to affect postoperative gut recovery were controlled, with the only exception being the administration of the anticholinergic glycopyrrolate and the acetylcholinesterase inhibitor neostigmine at the end of the operation. Although a fixed dose of neuromuscular reversal was administered, further comparison of glycopyrrolate mcg.kg^−1^ and neostigmine mg.kg^−1^ with OCTT did not reveal any correlation. The single dose of these two agents was so small that it is unlikely to have produced a clinically significant sustained effect in the postoperative period. All patients of this study participated in a structured postoperative programme that included early oral intake, early mobilisation and reduction of postoperative administration of IV fluids, with postoperative analgesia aimed to achieve a VAS score of 5. Although patients in the Ep-R group had large variations in VAS score, no correlation was found between pain scores and GI motility.

The difficulty in comparing results from different studies on GI motility is increased by problems of methodology and design, lack of reporting the level of sensory block and failure to control factors known to affect the GI tract. Clinical assessment of the overall GI motility also presents difficulty. The time to first bowel sounds is not a specific test, while the time to passage of first flatus, although a measure of coordinated bowel function, is an insensitive marker, as patients usually overlook the sensation when influenced by wound pain and analgesic medications [17]. Whilst the time to the first passage of stools represents a clear clinical endpoint, it may indicate only distal bowel emptying and not the function of the entire gut [1, 17]. Other tests, such as the scintigraphic imaging investigations of radio-labelled meals used for the assessment of GI recovery have been shown to be relatively independent of the clinical markers used to evaluate resolution of the ileus [15, 17, 18]. For this reason, in the present study we assessed GI motility not only by clinical signs of bowel recovery (flatus, bowel sounds, defecation), but also by measuring the OCTT and the migration of radiopaque markers by abdominal radiographs. It is noteworthy that our findings showed no correlation between the clinical bowel signs and the OCTT and CTT data.

The lactulose H_2_-breath test is a simple, non-invasive method widely used for quantifying the OCTT, where the substance (lactulose) and dose (10 g) used in a controlled diet setting improves the reliability of the test [18]. In addition, testing subjects preoperatively allowed identification of potential, pre-existing gut abnormalities (H_2_-non producers) and confirmed the homogeneity of the cohort. The main limitation of the second objective test used, the CTT, is the physiological variability in colonic movement. The preoperative administration of 20 radiopaque markers allows for the evaluation of the baseline colonic transit time with an exponential rate of disappearance of the markers. The mean number of markers retained in the healthy colon 3 days after indigestion is reported as 2 [19]. In the present study although there was a difference between preoperative and postoperative CTT, no difference was shown between groups. This may be because we performed only one X-ray (rather than a series of daily X-rays) in order to minimise radiation exposure and discomfort to our patients or that the study was underpowered to detect the difference.

Several other limitations must be acknowledged. The study was monocentric and half blinded. Although there were no demographic differences between the groups, the study included mainly men (men 27: women 3). Women have slightly different rates of gut motility especially regarding the CTT. Also, the study was underpowered to assess the secondary outcomes (VAS) scores which could have an effect on gut motility.

## Conclusions

The present study showed that objective tests are capable of detecting the delayed motility of the whole GI system on the first and third postoperative days after thoracic surgery. It also demonstrated that continuous epidural analgesia with ropivacaine or combination of ropivacaine and morphine improved GI motility in comparison to intravenous morphine. This is particularly important following thoracic surgery, as these patients are prone to pulmonary complications, which can be exacerbated by postoperative ileus.

## Abbreviations

ANOVA: Analysis of variance
ASA: American society of anesthesiologists
CTT: Colonic transit time
GI: Gastrointenstinal
GLM: Generalised linear model
IV: Intravenous
LAs: Local anesthetics
MMC: Migrating myoelectric complex
OCTT: Orocaecal transit time
PCA: Patient controlled analgesia
POI: Postoperative ileus
TEA: Thoracic epidural analgesia
VAS: Visual analogue scale
